# Supplementary material for: DNA nanomapping using CRISPR-Cas9 as a programmable nanoparticle
Source: Nat Commun. 2017 Nov 21;8:1665. doi: 10.1038/s41467-017-01891-9 (PMC5698298; doi:10.1038/s41467-017-01891-9)
Supplement: Supplementary file 1 — Supplementary Information [file 41467_2017_1891_MOESM1_ESM.pdf]

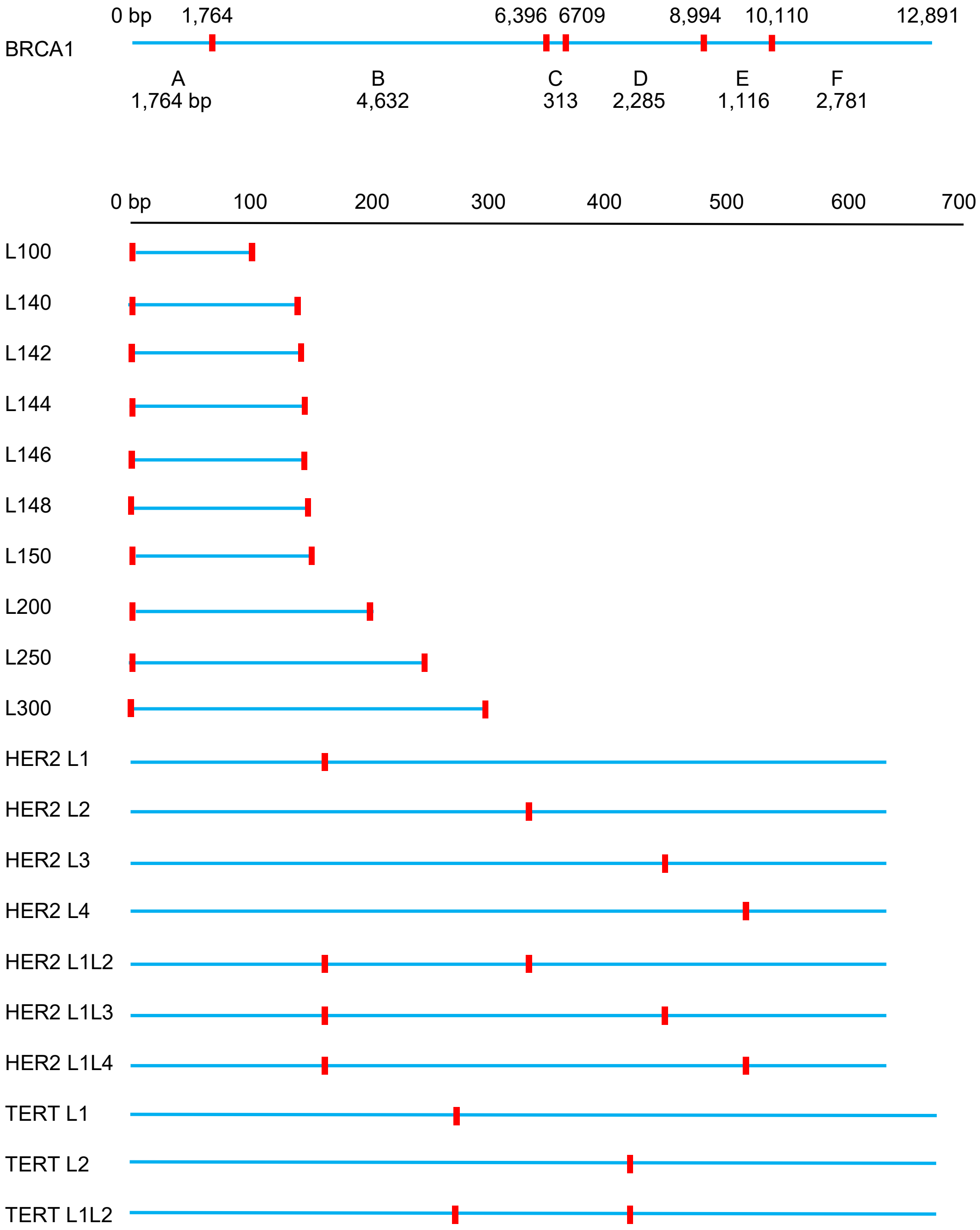

Supplementary Figure 1. DNA constructs. Red hash marks show the location of the GG PAM sequence within the sgRNA binding site for each construct. Molecules are depicted with the 5' end to the left. For clarity, only the sites with perfect matches to the *Alu* sgRNA are shown for the *BRCA1* amplicon; see **Supplementary Table 3** for the locations of mismatch sites.

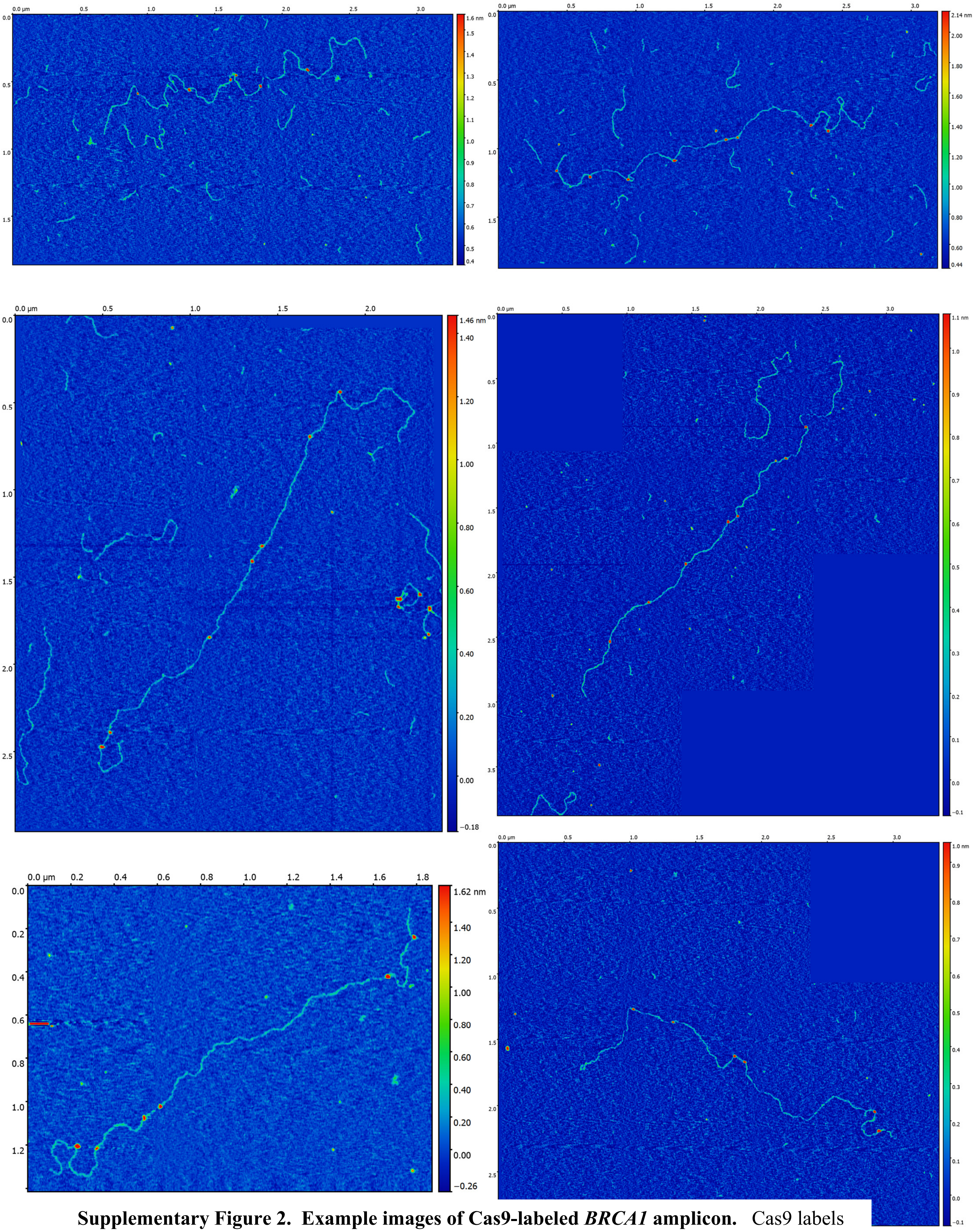

Supplementary Figure 2. Example images of Cas9-labeled *BRCA1* amplicon. Cas9 labels appear in red on the DNA backbone. Images larger than 2um x 2um are composed of 2 or more single frames.

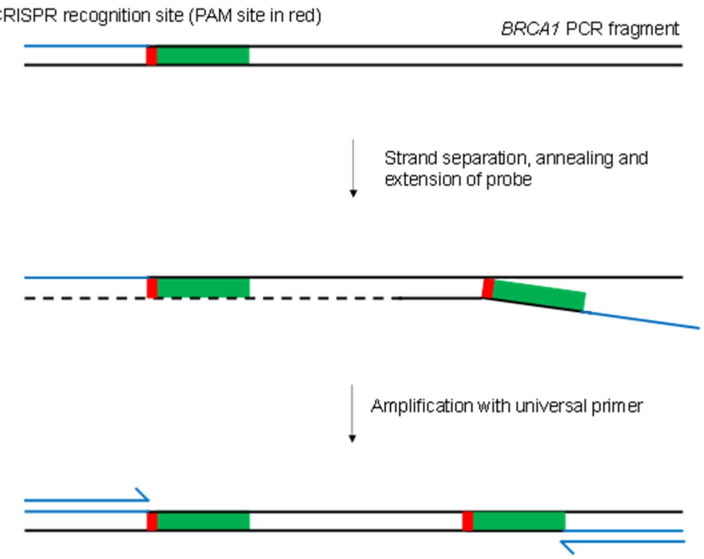

Supplementary Figure 3. Scheme for creating ladder constructs.

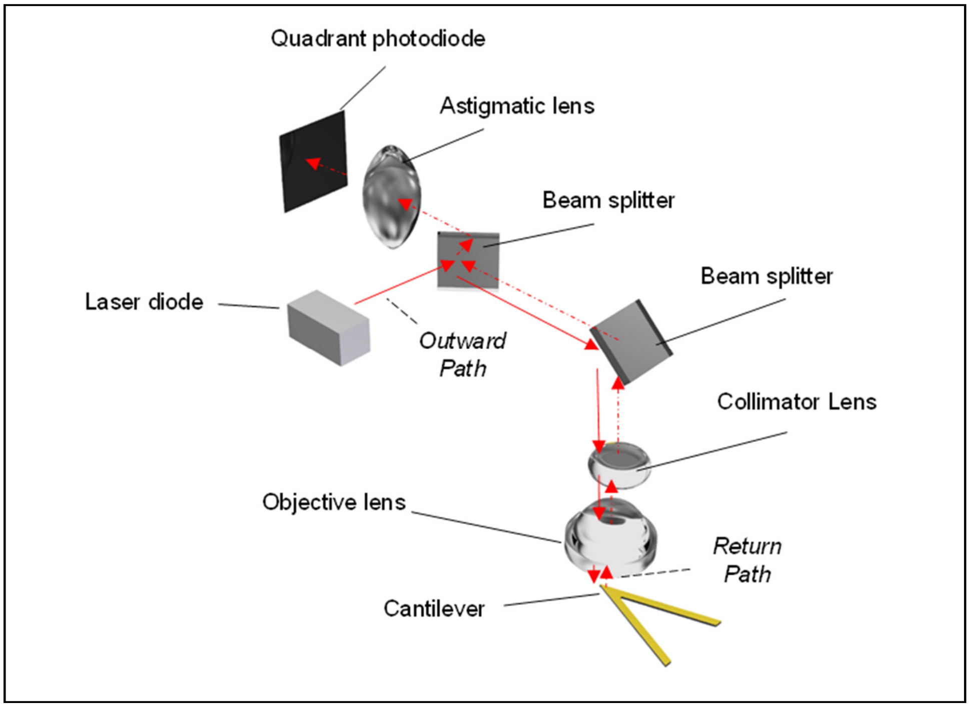

Supplementary Figure 4. Schematic of the optical elements in the DVD optical pickup unit.

Amplicon(s)	Guide RNA(s) Site	Guide RNA Concentraton (final), nM	Type of CRISPR	Number of Molecules Analyzed	Labeling Efficiency	Percent of Outliers
TERT L1	275 bp	400	WT Cas9	508	99%	2%
TERT L2	420 bp	400	WT Cas9	505	94%	5%
HER2 L1	164 bp	400	WT Cas9	501	96%	6%
HER2 L2	335 bp	400	WT Cas9	504	94%	3%
HER2 L3	451 bp	400	WT Cas9	505	91%	8%
HER2 L4	498 bp	400	WT Cas9	506	98%	6%
TERT L1 + L2	275 bp + 420 bp	400 + 400	WT Cas9	502	88%	5%
HER2 L1 + L2	164 bp + 335 bp	400 + 400	WT Cas9	501	84%	6%
HER2 L1 + L3	164 bp + 451 bp	400 + 400	WT Cas9	499	86%	5%
HER2 L1 + L4	164 bp + 498 bp	400 + 400	WT Cas9	504	90%	5%
HER2 L1 + L3	164 bp + 451 bp	400 + 400	Cas9 Nickase	503	68%	4%
HER2 L1 + L4	164 bp + 498 bp	400 + 400	Cas9 Nickase	506	95%	5%

Supplementary Table 1. Cas9 labeling efficiency. The labeling efficiency was then determined based on the number of sites per molecule, as represented in the sixth column. Molecules not within +/- 15% of the expected length, using an assumed DNA pitch of 0.34nm/bp, were excluded from analysis. The percent of outliers represented in the last column was determined by the statistical procedure found in the Methods.

Gene Targeted	Name	Sequence
**Primers:**		
TERT	T680-f	AGGTCGGAACGAGGGTCACG
TERT	T680-r	TGTCCTCACGCTCTCGGGTG
HER2	H645-f	GGCCAGAGGACGAGTGTGGT
HER2	H645-r	CTCAGGGTGGCACGGCAAAC
BRCA1	BRCA1-f	ACCCCAACATTGATTCCTTTC
BRCA1	BRCA1-r	CACAGGGAGAAAGTCTGCAAG
Ladder	Ladder-Primer	GTGATCCACCTGCCTCGG
**Probes:**		
Ladder-Probe-75bp		
GTGATCCACCTGCCTCGGTGTAATCCCAGCACTTTGGGAGGAACAAATCATCAAGAAATGATGGGC		
Ladder-Probe-100bp		
GTGATCCACCTGCCTCGGTGTAATCCCAGCACTTTGGGAGGTGACTGCCCCAGCATAC		
Ladder-Probe-150b		
GTGATCCACCTGCCTCGGTGTAATCCCAGCACTTTGGGAGGACTTTTTCCCTCTAACTCTTTTTCCC		
Ladder-Probe-200bp		
GTGATCCACCTGCCTCGGTGTAATCCCAGCACTTTGGGAGGGGGATGGGTAAGGATTTGAGAACTGC		
Ladder-Probe-250bp		
GTGATCCACCTGCCTCGGTGTAATCCCAGCACTTTGGGAGGTCAGAAGTCTTCTCTGCCCACATACC		
Ladder-Probe-300bp		
GTGATCCACCTGCCTCGGTGTAATCCCAGCACTTTGGGAGGGTGAGCAAGACTGGCACCTG		
**sgRNAs:**		
TERT	275 bp	r(GTAUCCCUGGAGCUUCGGUCTGG)
TERT	420 bp	r(CACACUCCUCCCAGAGCGGCCGG)
HER2	164 bp	r(GCUAGAGAAGCCAUGCCAAGAGG)
HER2	335 bp	r(UCCCUGGUUCACUUGGACCUGGG)
HER2	451 bp	r(UCAACUGCAGCCAGUUCCUUCGG)
HER2	521 bp	r(CGAGUACUGCAGGGGUAUGAGGG)
BRCA1	Alu-sgRNA	r(UGUAAUCCCAGCACUUUGGGAGG)

Supplementary Table 2. Primer and sgRNA sequences.

Target sequence	Location from 5' end	Number of mismatches to sgRNA
CCTCCTAAAGTGCTGGGATTACA	495	1
CCTCCCAAAGTGCTAGGATTACA	1457	1
CCTCCCAAAGTGCTGGGATTACA	1764	0
CCTTCCAAAGTGCTGGGATTACA	3301	1
CCTCCCAAAGTGCTAGGATTACA	4818	1
CCTCCCAAAGTGCTGGGATTACA	6418	0
CCTCCCAAAGTGCTGGGATTACA	6731	0
CCTCCCAAAGTGCTGGAATTATA	7835	2
CCTCCCAAAGTGCTGGGATTACA	9013	0
CCTCCCAAAGTGCTGGGATTACA	10132	0

Supplementary Table 3. ***Alu*** **target sequences in** ***BRCA1*** **amplicon.** The location of the mismatch to the sgRNA sequence is indicated by an underscore.

BRCA1

Segment	Actual bp	Measured nm*	σ nm	μ calc bp	σ calc bp	Err bp	Abs err bp	CI95% bp	N
A	1,764	601.4	49.2	1,768.7	115.2	4.7	4.7	100.9	5
B	4,632	1,581.1	15.1	4,650.4	207.4	18.4	18.4	181.8	5
C	313	109.0	29.6	320.6	27.7	7.6	7.6	18.1	9
D	2,285	813.9	9.4	2,393.9	87.1	108.9	108.9	60.4	8
E	1,116	400.5	70.5	1,177.8	44.4	61.8	61.8	32.9	7
F	2,781	971.6	39.2	2,857.7	144.8	76.7	76.7	100.3	8
**Median**	2,025				101.1		40.1	**N total**	42
**High**	4,632				207.4		108.9		
**Low**	313				27.7		4.7		

Supplementary Table 4. ***BRCA1* amplicon measurement detail.** The nm-to-bp conversion assumes the solution value for DNA pitch (2.94 bp/nm). *Lengths correspond to segments A-F between Cas9 labels (see **Supplementary Figure 1**).

Ladder

Amplicon	Actual bp	Measured nm*	σ nm	μ calc bp	σ calc bp	Err bp	Abs err bp	CI95% bp	N
L100	100	34.4	4.8	101.1	14.2	1.1	1.1	1.3	475.0
L100	100	34.0	3.8	100.1	11.2	0.1	0.1	0.7	909.0
L100	100	34.6	4.3	101.9	12.6	1.9	1.9	0.7	1,135.0
L100	100	34.2	4.2	100.5	12.3	0.5	0.5	1.0	589.0
L100	100	34.0	4.3	100.0	12.5	0.0	0.0	0.9	760.0
L100	100	33.9	3.6	99.8	10.7	(0.2)	0.2	0.7	820.0
L150	150	51.1	5.5	150.3	16.3	0.3	0.3	0.5	4,470.0
L150	150	51.6	6.7	151.8	19.8	1.8	1.8	0.6	4,092.0
L150	150	51.7	5.3	152.0	15.7	2.0	2.0	0.5	3,346.0
L150	150	51.4	6.6	151.2	19.5	1.2	1.2	0.6	4,286.0
L150	150	51.0	6.3	150.0	18.6	0.0	0.0	0.6	4,359.0
L150	150	51.3	6.3	150.8	18.5	0.8	0.8	0.5	4,367.0
L200	200	66.9	8.4	196.9	24.6	(3.1)	3.1	1.3	1,284.0
L200	200	67.8	6.5	199.4	19.1	(0.6)	0.6	0.7	2,630.0
L200	200	66.9	7.1	196.6	21.0	(3.4)	3.4	1.0	1,643.0
L250	250	84.2	9.9	247.7	29.2	(2.3)	2.3	0.9	3,899.0
L250	250	83.3	7.4	245.0	21.9	(5.0)	5.0	0.9	2,148.0
L250	250	84.8	9.0	249.5	26.4	(0.5)	0.5	1.9	736.0
L300	300	100.5	11.6	295.7	34.0	(4.3)	4.3	1.6	1,647.0
L300	300	101.3	10.6	298.1	31.2	(1.9)	1.9	1.7	1,304.0
L300	300	100.1	12.8	294.3	37.5	(5.7)	5.7	1.8	1,606.0
L300	300	99.5	11.0	292.7	32.3	(7.3)	7.3	2.2	843.0
**Median**	150				19.3		1.5	**N total**	47,348.0
**High**	300				37.5		7.3		
**Low**	100				10.7		0.0		

Supplementary Table 5. Ladder amplicon measurement detail. The nm-to-bp conversion assumes the solution value for DNA pitch (2.94 bp/nm). *Lengths measured between Cas9 labels.

Ladder (140-150 bp)

Amplicon	Actual bp	Measured nm*	σ nm	CI95% nm	N
L140	140	47.2	6.9	0.12	13,760
L142	142	47.8	5.9	0.11	12,133
L144	144	48.3	6.1	0.08	21,697
L146	146	49.8	7.2	0.12	14,136
L148	148	50.1	7.1	0.14	9,483
L150	150	51.0	6.7	0.09	20,305
**Median**	145			**N total**	91,514
**High**	150				
**Low**	140				

Supplementary Table 6. Fine ladder measurement detail. *Lengths measured between Cas9 labels.

Amplicon	Actual bp	Measured nm*	σ nm	μ calc bp	σ calc bp	Err bp	Abs err bp	CI95% bp	N
TERT L1	275	91.9	13.1	270.2	38.6	(4.8)	4.8	3.4	481
TERT L2	420	142.5	14.1	419.0	41.3	(1.0)	1.0	3.8	460
HER2 L1	164	58.0	9.3	170.5	27.3	6.5	6.5	2.5	462
HER2 L3	451	153.9	18.8	452.4	55.3	1.4	1.4	5.1	450
HER2 L4	521	175.0	18.4	514.6	54.1	(6.4)	6.4	4.9	474
TERT L1L2, L1	275	93.2	9.6	274.1	28.3	(0.9)	0.9	2.7	408
TERT L1L2, L2	420	144.8	12.7	425.7	37.3	5.7	5.7	3.6	408
HER2 L1L2, L1	164	57.8	9.9	169.8	29.1	5.8	5.8	2.8	404
HER2 L1L2, L2	335	113.3	11.3	333.1	33.3	(1.9)	1.9	3.2	404
HER2 L1L3, L1	164	57.2	9.2	168.1	27.2	4.1	4.1	2.6	432
HER2 L1L4, L4	521	177.6	17.9	522.1	52.5	1.1	1.1	5.0	432
**Median**	335				37.3		4.1	**N total**	4,815
**High**	521				55.3		6.5		
**Low**	164				27.2		0.9		

Supplementary Table 7. *TERT* and *HER2* amplicon measurement detail. The nm-to-bp conversion assumes the solution value for DNA pitch (2.94 bp/nm). * Lengths to Cas9 labels measured from the DNA 5' end.

Amplicon	Template	Primer 1	Primer 2
BCL2-IGH fusion	Clinical samples and cell line DOHH2	MBR/02	Eμ/01
Wild type *BCL2*	Human Genomic DNA	MBR/02	MBR-r1
Wild type *IGH*	Human Genomic DNA	Eμ/01-1f	Eμ/01

Supplementary Table 8. Primer nomenclature for BCL2/IGH fusion experiments.

Gene Targteted	Name	Sequence
**Primers**:		
BCL2	MBR/02	CTATGGTGGTTTGACCTTTAGAGAGTTGCTTTACG
BCL2	MBR-r1	TGTCCCTACCAACCAGAAGGTTGTC
IGH	Eμ/01-1f	TTGAGGGCTGGGGTCTCCCGCGTGT
IGH	Eμ/01	CTAGGCCAGTCCTGCTGACGCCGCATCGGTGATTC
**sgRNA targets (PAM site highlighted)**:		
BCL2	BCL2-MBR-L	CACGTAAAGCAACTCTCTAAAGG
BCL2	BCL2-MBR-R	TAAAAATTAGAATCATTCAAAGG
IGH	J145	ACCCTGGTCACCGTCTCCTCAGG
IGH	J2	ACCCTGGTCACTGTCTCCTCAGG
IGH	J3	ACAATGGTCACCGTCTCTTCAGG
IGH	J6	ACCACGGTCACCGTCTCCTCAGG

Supplementary Table 9. Primer sequences for BCL2/IGH fusion experiments.
